# Chemical composition, anticancer, antimicrobial activity of *Aloysia citriodora* Palau essential oils from four different locations in Palestine

**DOI:** 10.1186/s12906-024-04390-9

**Published:** 2024-02-16

**Authors:** Nawaf Al-Maharik, Yousef Salama, Nisreen Al-Hajj, Nidal Jaradat, Naji Thaer Jobran, Ismael Warad, Lina Hamdan, Moataz Abo Alrob, Asil Sawafta, Adel Hidmi

**Affiliations:** 1 Department of Chemistry, Faculty of Sciences, An-Najah National University, Nablus P.O. Box. 7, Nablus, 99900800 Palestine; 2 An-Najah Center for Cancer and Stem Cell Research, Faculty of Medicine and Health Sciences, An-Najah National University, P.O. Box 7, Nablus, 00970 Palestine; 3 Department of Pharmacy, Faculty of Medicine and Health Sciences, An-Najah National University, Nablus, P.O. Box. 7, Palestine; 4 Department of Chemistry, Faculty of Sciences, Birzeit University, Birzeit, P.O. Box. 7, Palestine

**Keywords:** *Aloysia citriodora*, Phytochemicals, Colorectal cancer, Antibacterial, Antifungal

## Abstract

The primary aim of this investigation was to determine the anticancer and antimicrobial properties of essential oils (EOs) extracted from the leaves of *Aloysia citriodora* Palau, which were procured from four separate locations in Palestine, in addition to analyzing their chemical composition. These areas include Jericho, which has the distinction of being the lowest location on Earth, at 260 m below sea level. The EOs were acquired by hydrodistillation, and their chemical composition was examined utilizing gas chromatography-mass spectrometry (GC-MS). The minimum inhibitory concentration (MIC) of EOs was assessed against six bacterial strains and one fungal species using 96-well microtiter plates. The primary components found in these oils are geranial (26.32–37.22%), neral (18.38–29.00%), and *α*-curcumene (7.76–16.91%) in three regions. *α*-Curcumene (26.94%), spathulenol (13.69%), geranial (10.79%), caryophyllene oxide (8.66%), and neral (7.59%) were found to be the most common of the 32 chemical components in the EO from Jericho. The EOs exhibited bactericidal properties, particularly against *Staphylococcus aureus*, methicillin-resistant *Staphylococcus aureus* (MRSA), and showed highly effective fungicidal activity. Nevertheless, the antifungal efficacy of the EO was found to surpass its antibacterial activity when administered at lower dosages. The EOs exhibited anticancer activities against melanoma cancer cells, as indicated by their IC_50_ values, which ranged from 4.65 to 7.96 μg/mL. *A. citriodora* EO possesses substantial antifungal and anticancer characteristics, rendering it appropriate for utilization in food-related contexts, hence potentially enhancing the sustainability of the food sector.

## Introduction

In recent years, natural products have garnered increased attention from the general public owing to their distinctive biological characteristics and purported absence of adverse effects. Consequently, researchers have directed their endeavors towards establishing empirical support for the therapeutic effectiveness of these products in mitigating a diverse array of pathological conditions, including microbial infections and different forms of malignancy. Research has demonstrated that phytochemicals can change the gene expression profiles of cancer cells, decreasing their survival and impeding their migratory capabilities [1–5]. The pharmaceutical and food sectors have greatly benefited from the increased discovery of natural compounds with biological significance. Phytochemicals, known as essential oils (EOs), are of paramount importance in the defense mechanisms of plants. EOs are highly concentrated hydrophobic liquids composed of volatile terpenes, which are present in different amounts and encompass a wide array of chemical structures [6]. Pharmaceutical formulations commonly integrate EOs or utilize them in aromatherapy due to their diverse health benefits, which include antibacterial [7], antiviral [8], antibacterial [9], antioxidant [9], anticancer [9], anti-inflammatory [10], insecticidal [11], and immunomodulatory properties [12]. Recent studies have focused on the synergistic and antagonistic activity of particular EOs and their major and minor constituents in preventing or inducing cancer cell death via several signalling pathways [13]. According to the literature, *Aloysia citriodora* EO has a variety of compounds, including neral, geranial, limonene, *α*-curcumin, and others, all of which have anticancer and antimicrobial properties [14].

*Aloysia citriodora* Palau, known as lemon verbena, is a perennial plant native to South America, specifically Argentina and Chile [14–16]. Increasing consumer demand has driven the expansion of lemon verbena cultivation to regions beyond its indigenous range in Argentina and Chile, such as Europe and the Africa-Mediterranean areas [14–17]. The EO obtained from the leaves of this plant, with its aromatic and medicinal properties, is a welcome addition to many different kinds of food and drink, from infusions to non-alcoholic beverages, making it a prominent contender in the global herbal market [14–18]. The pharmaceutical industry uses lemon verbena for its diuretic, antispasmodic, and sedative properties [14–19]. Traditionally, infusions made from the leaves of this plant have been employed to alleviate many symptoms, including indigestion, fever, asthmatic spasms, bloating, cramping, diarrhea, restlessness, anxiety, discomfort, and insomnia [14, 15, 19].

Multiple scholarly inquiries have been carried out to analyse the chemical composition of *A. citriodora* EO obtained from different parts of the plant, with a particular focus on its potent and appealing aromatic properties [15–20]. The published literature enlightens the differences seen in the composition of EOs extracted from *A. citriodora*, with oxygenated monoterpenes being the most common components.

The majority of literature discussing the compositions of *A. citriodora* EO adheres to a similar pattern, wherein oxygenated monoterpenes, such as geranial, neral, and limonene, are identified as the prevailing class. However, certain profiles deviate from this pattern, mainly consisting of oxygenated sesquiterpenes such as spathulenol and caryophyllene oxide [14, 19]. The quality and quantity of EO are influenced by various factors, including genetic makeup, environmental conditions, morphogenetic variability of plant components, ontogenetic variability during developmental phases, and diurnal temperature fluctuations [18].

Our current research studies the chemical compositions and pharmacological characteristics of EOs derived from medicinal plants found in Palestine. Specifically, we focus on the plants growing near the Dead Sea. The area is distinguished by its saline soil, dry summers, and scarce rainfall. As part of our inquiry, we have acquired *A. citriodora* Palau EO from four different areas in Palestine, including Jericho. This study analyzes the chemical composition of the collected EOs, along with an evaluation of their antimicrobial and anticancer properties.

## Materials and methods

### Plant materials and essential oils distillation

The collection of leaves of cultivated *A. citriodora* took place in June 2022 across four distinct governorates in Palestine: Jericho, Jerusalem, Hebron, and Qalqilya. Our specialist Professor Nidal Jaradat identified the plant leaves, and voucher specimens were stored at the Herbal Products Laboratory at An-Najah National University, where they were assigned the collective code Pharm-PCT-2780A-D. Following rinsing with ionized water to remove the dust, the leaves were dried in a shaded location at room temperature (25 ± 3 °C) and humidity (55 ± 4 RH). The dried leaves were mechanically crushed into tiny pieces to assist in the extraction of the EO. The dried leaves were mechanically crushed into tiny fragments to make it easier to extract the essential oil. 100 Grams of powdered leaves and 100 ml of distilled water were placed in a Clevenger apparatus (Merck, USA) and heated to 100 degrees Celsius for 3 hours. The acquired essential oil (EO) was dehydrated using anhydrous Na2SO4 and stored in sealed containers in a dark atmosphere at a temperature of 4 °C until it was used. We conducted each experiment twice.

### Qualitative and quantitative analysis of the extracted oils

The chemical composition of the extracted oils was analyzed using Gas Chromatography-Mass Spectrometry (GC–MS) with the Perkin Elmer Clarus 500 GC, which is connected to a Perkin Elmer Clarus 560 mass spectrometer. Separation of the samples was achieved by using Perkin Elmer Elite-5-MS fused silica capillary columns (film thickness 0.25 m, 30 m × 0.25 mm). The column temperature increased gradually from 50 °C to 280 °C, with a rate of 4 °C per minute. The carrier gas used was helium, which maintained a steady flow rate of 1 ml/min throughout the chromatographic run. One μL of the tested oils was dissolved in acetonitrile and injected in split mode at 250 °C using a split ratio of 1:50. The transfer line and ion source were both adjusted to a temperature of 250 °C. The mass spectra were acquired using electron ionization (EI) mode, with an ionization voltage of 70 eV. The mass spectra were recorded throughout a scan duration of 62.5 minutes, covering the mass-to-charge ratio (m/z) range of 50 to 500.

The identification of EO components was achieved by comparing the estimated temperature-programmed retention indices, as well as the mass spectra and fragmentation patterns, with those documented in the literature (NIST). Kovats retention indices (RI) were calculated using linear interpolation in relation to the retention durations of a series of n-alkanes (C_6_-C_30_).

### Chemicals

DMSO, MTT (3-(4,5-Dimethylthiazol-2-yl)-2,5-Diphenyltetrazolium Bromide) and fluorescein isothiocyanate (FITC)–phalloidin were purchased from Sigma-Aldrich (St. Louis, MO, USA).

### Cell lines

The human embryonic kidney cells [HEK-293] (ATCC CRL-1573); American Type Culture Collection (ATCC), Manassas, VA, USA], A549 (ATCC CCL-185), A-431 (ATCC CRL-1555) cell lines were maintained in Dulbecco’s Modified Eagle’s Medium (DMEM, high-glucose containing L-glutamine, phenol red (Fuji Film Wako, Osaka, Japan), 10% fetal bovine serum (FBS; G.E. Healthcare, Chicago, IL, USA), and 1% penicillin/streptomycin (P/S) (Nacalai Tesque, Kyoto, Japan). Human HS-5 stromal cultures were maintained in Iscove’s Modified Dulbecco’s Medium (IMDM; Wako, Japan), containing 10% FBS and 1% P/S. Human HT29 (ATCC; HTB-38), HCT 116 (ATCC CCL-247), SW620 [SW-620 (ATCC CCL-227), SW480 [SW-480] (ATCC CCL-228) cells were cultured in McCoy’s 5a Medium Modified, Wako, Japan) containing 10% FBS and 1% P/S. Human umbilical vein endothelial cells (HUVECs; ATCC, CRL-1730) were cultured at 37 °C/5% CO_2_ on 0.1% gelatin (Wako Pure Chemicals)-coated culture plates (Falcon) in endothelial growth medium-2 (EGM-2; Lonza; cc4176). K-562 (ATCC CCL-243) cells were cultured in RPMI-1640 containing 10% FBS and 1% P/S at 37 °C/5% CO_2_.

### Quantitative reverse transcriptase-polymerase chain reaction (qPCR)

The extraction of total RNA from cell lines was performed using TRIzol reagent, followed by reverse transcription into complementary DNA (cDNA) using the PrimeScript RT Master Mix, in accordance with the instructions provided by the manufacturer. The cDNA was kept at a temperature of − 30 °C. The qPCR experiments were conducted in duplicate and replicated independently twice. The primers utilized in this study are as follows: Forward; Reverse.

h-MMP9 5′-GGACTCGGTCTTTGAGGAGC-′3; 5′-.

CCTGTGTACACCCACACCTG-′3.

h-β-actin 5′-AGACCTGTACGCCAACACAG-′3; 5′-.

TTCTGCATCCTGTCGGCAAT-′3.

### Cell cultures with EOs

HT-29 cells (2105 cells/well) were inoculated into 6-well plates (TPP, Switzerland) and incubated overnight prior to the addition of DMSO (control) and *A. citriodora* EOs at concentrations ranging from 5 to 50 μg/mL. EOs were applied to proliferation assays at a concentration of 25 μg/mL if not otherwise specified. Cell viability was assessed after 24 hours by employing trypan blue (cat. 207–17,081; Fuji Film Wako), whereas cell proliferation was evaluated utilizing the MTT test kit (Sigma, St. Louis, MO, USA).

### Webserver GEPIA 2

GEPIA 2 (http://gepia2.cancer-pku.cn/#index) is a comprehensive web server and resource for comprehensively studying the link between gene expression across various cancer types [21].

### Antimicrobial activity

The antimicrobial activity of *A. citriodora* EOs was assessed using a fungal strain, *Candida albicans* (American type culture collection (ATCC 90028), and six bacterial strains: *Escherichia coli* (ATCC 25922), *Klebsiella pneumonia* (ATCC 13883), *Proteus vulgaris* (ATCC 8427), *Pseudomonas aeruginosa* (ATCC 9027), *Staphylococcus aureus* (ATCC 25923), and methicillin-resistant *Staphylococcus aureus* (MRSA) that was confirmed through diagnostic methods. The EOs were assessed for their antibacterial activity using the broth microdilution technique, following the previously reported method [22]. A stock solution with a concentration of 50 mg/mL was prepared by combining each EO with 20% DMSO and 60% Muller–Hinton broth. The EO solutions that were generated were serially diluted by a factor of two in sterile Muller-Hinton broth to obtain final concentrations of 25.00, 12.50, 6.25, 3.125, etc., mg/mL (RPMI medium was used for the *C. albicans* strain). The DMSO concentration in the first well was 5% and was subsequently diluted twofold in order to eliminate any potential antimicrobial effect. Aseptically, the dilution procedure was executed on 96-well plates. The microbes that were being studied were injected aseptically into wells 1–11, while the EO was introduced into wells 1–10. Micro-well 12 (devoid of essential oil and microbes) was utilized as a negative control for microbial proliferation, whereas micro-well 11 served as the positive control. Plates containing test bacterial strains were incubated at 35 °C for a duration of 18–24 hours, while plates containing *Candida albicans* were incubated at the same temperature for 48 hours. The MIC of the tested EOs was determined by calculating the lowest concentration of EO in the micro-well at which no apparent microbiological growth occurred. Ciprofloxacin and ampicillin were used as positive antibacterial activity controls in this investigation. As a positive control for antifungal activity, fluconazole was used. While all of the studies that did not use plant material were considered negative controls. The samples’ antibacterial activity was evaluated in triplicate.

### Statistical analysis

Each experiment was conducted in triplicate. In statistics, the mean and standard deviation are presented. Following the analysis of variance or student’s t-test, Tukey HSD post-hoc tests were conducted. *P*-values less than 0.05 were deemed to indicate significance.

## Results and discussion

**Fig. 1:**
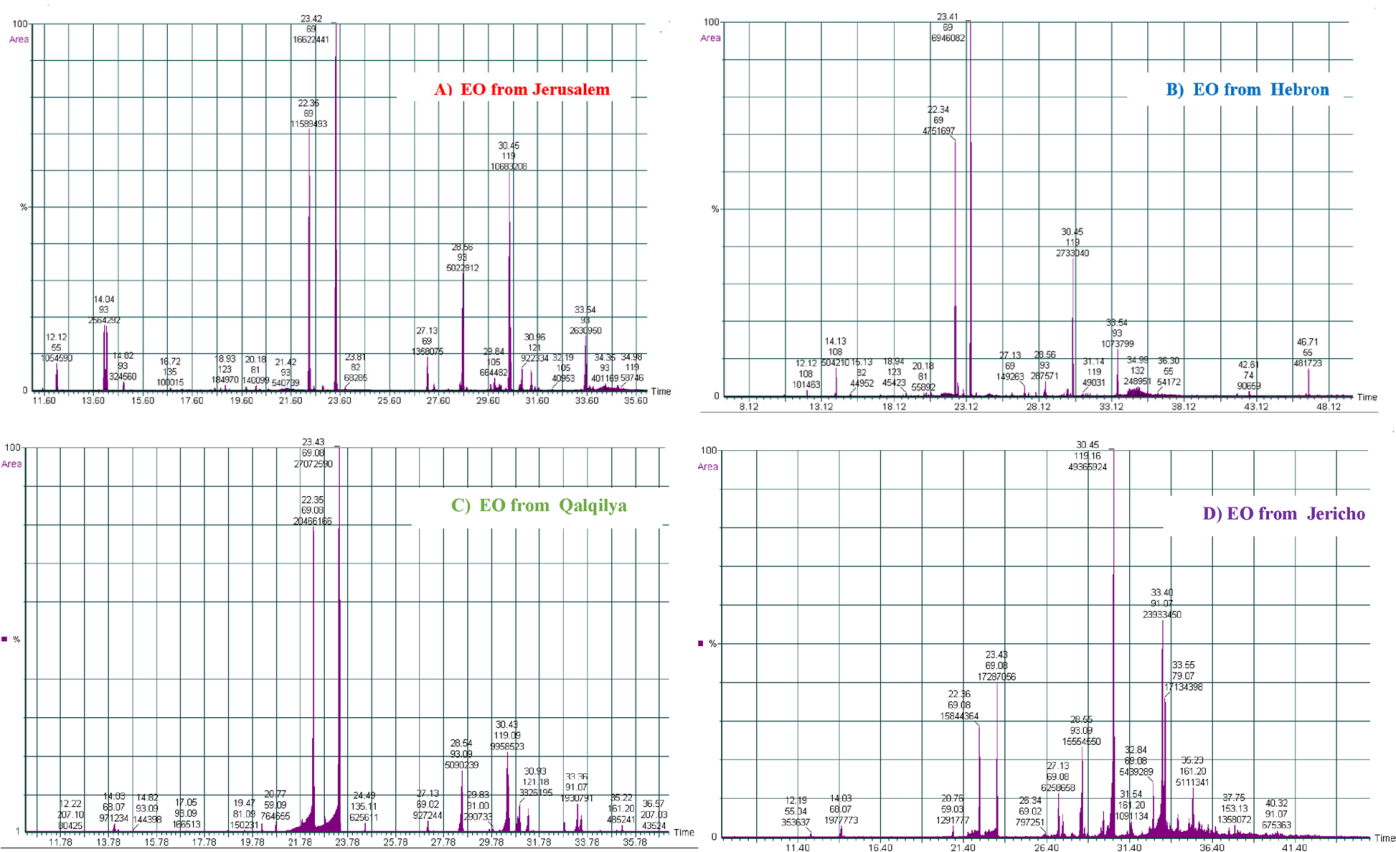
Gas chromatography chromatograms of essential oil of *A. citriodora* leaves obtained from four diverse localities of Palestine, namely Jerusalem, Hebron, Jericho, and Qalqilya

Further investigation is necessary to explore the phytochemical, toxicological, and pharmacological characteristics *of A. citriodora*, given its extensive utilization in traditional medicine and the acknowledgment of its products by the pharmaceutical industry. The chemical composition of EOs derived from *A. citriodora* collected from four sites in Palestine was examined through the utilization of GC-MS, as depicted in Fig. 1. Table 1 displays the names, retention times (RT), retention indices (RI), and percentages of recognized constituents for the EOs. The hydrodistillation of *A. citriodora* leaves produced light yellow EOs with yields of 0.92, 0.86, 0.45, and 0.36%, respectively, from Qalqilya, Jerusalem, Hebron, and Jericho.

**Table 1:** Phytochemical composition of *A. citriodora* essential oil collected from four locations in Palestine

No.	Compounds	Content%				RI	RT
		Jericho	Qalqilya	Hebron	Jerusalem		
1	4,4-Dimethyl-2-butenolide	–	–	0.17	–	945	10.45
2	Sabinene	–	–	–	0.14	970	11.55
3	6-Methylhept-5-en-2-one	0.22	–	0.54	1.67	984	12.20
4	Limonene	0.74	0.86	0.21	4.06	1026	14.03
5	1,8-Cineole	0.09	0.32	2.70	3.96	1030	14.19
6	cis-Ocimene	–	0.05	–	0.02	1034	14.38
7	trans-Ocimene	–	0.22	–	0.51	1045	14.82
8	ND	–	–	0.02	–	1051	15.07
9	3-Methylcyclohex-2-en-1-one	–	–	0.24	0.19	1052	15.13
11	cis-Sabinene hydrate	0.06	0.12	–	–	1068	15.85
12	cis-4-Thujanol	–	0.28	–	–	1097	17.05
13	α-Thujone	–	–	0.17	0.12	1101	17.19
15	6-Camphenol	–	–	–	0.04	1107	17.42
16	cis-*p*-Menth-2-en-1-ol	–	–	0.05	–	1023	18.08
17	1-Terpineol	–	–	0.07	0.17	1135	18.52
19	exo-Isocitral	–	–	0.15	–	1140	18.74
20	γ-Geraniol	–	–	–	0.12	1140	18.74
21	Veratrole other	–	–	0.24	–	1145	18.93
22	Sabina ketone	–	–	0.11	–	1155	19.32
23	Rosefuran epoxide	–	–	0.10	–	1167	19.79
24	*β*-Pinene oxide	–	–	–	0.29	1145	18.94
25	Ethyl chrysanthemate	–	–	–	0.05	1149	19.09
26	Citronellal	–	–	–	0.21	1167	19.79
28	Isogeranial	0.06	0.91	0.30	0.22	1177	20.19
29	Ethyl-3(2-furyl)-propanoate	–	–	–	0.09	1181	20.335
30	Isomenthol	–	–	0.31	–	1181	20.34
31	cis-Dihydrocarvone	–	–	0.39	–	1190	20.68
32	4-Methylguaicol other	–	–	–	0.09	1190	20.68
33	α-Terpineol	0.76	1.06	–	–	1192	20.755
34	Isodihydro carveol	–	–	0.4	0.86	1210	21.415
36	Linalool formate	–	–	1.3	0.37	1216	21.66
37	cis-4-Thujanol acetate	–	–	–	0.07	1222	21.86
38	neoisoDihydro carveol	–	–	0.27	0.05	1225	21.99
40	Nerol	0.44	1.01	0.18	0.05	1228	22.076
42	Neral	7.59	29	25.46	18.35	1236	22.36
43	Carvone	–	–	1.22	0.23	1241	22.53
44	Geraniol	–	1.26	–	–	1249	22.82
45	Piperitone	–	–	0.62	0.30	1251	22.89
46	Methyl citronellate	0.49	–	–	–	1251	22.92
47	Geranial	10.79	37	37.22	26.32	1265	23.421
49	Citronellyl formate	–	–	0.33	0.11	1276	23.80
50	Thymol	–	0.96	–	–	1295	24.50
51	Ethyl nerolate	–	–	0.44	–	1348	26.26
52	Neric acid	0.22	–	–	–	1351	26.35
53	Geranyl acetate	–	–	0.8	–	1373	27.13
54	α-Ylangene	3.00	1.13	–	2.15	1374	27.133
55	Bourbonene	1.47	–	0.30	0.47	1382	27.38
57	Methyl eugenol	–	–	0.42	–	1395	27.89
58	Sesquithujene	0.25	–	–	0.07	1399	27.96
59	α-Cedrene	2.19	–	0.44	0.56	1415	28.45
61	*Β*-Caryophyllene	6.14	6.00	1.54	7.95	1419	28.55
62	*β*-Cedrene	0.63	0.16	–	0.29	1422	28.69
63	α-Caryophyllene S	0.62	0.1	0.31	0.38	1454	29.68
64	Allaromadendrene	1.68	0.41	–	1.05	1459	29.83
	ND				0.75	1465	30.034
65	10-*β*-Cadina-1(6),4-diene	–	–	–	0.12	1474	30.30
66	*α*-Curcumene	26.94	7.76	14.64	16.91	1479	30.45
67	*α*-Zingiberene	–	–	–	0.54	1492	30.88
69	Bicyclogermacrene	0.09	2.79	–	–	1494	30.94
70	δ-Acoradiene	–	–	–	1.46	1495	30.96
71	*β*-Bisabolene	0.33	–	–	–	1505	31.27
72	α-Cuprenene	–	2.31	–	1.28	1507	31.32
74	*α*-Amorphene	–	–	–	0.22	1512	31.48
75	Cubebol	–	0.23	–	–	1514	31.539
76	*δ*-Cadinene	0.98	–	–		1515	31.541
77	γ-Cadinene		–	–	0.18	1517	31.63
78	*α*-Cadinene	0.28	–	–	0.06	1536	32.18
79	ND	0.44	–	–	–	1551	32.61
80	E-Nerolidol	3.29	0.32	–	–	1558	31.84
82	Spathulenol	13.69	2.63	–	0.08	1577	33.40
83	Caryophyllene oxide	8.66	1.52	5.75	4.17	1583	33.56
84	Khusien-12-al	–	–	–	0.22	1588	33.71
85	anti-anti-anti-Helifolen-12-al B	–	–	–	0.18	1592	33.84
86	*β*-Atlantol OS	–	0.23	–	0.79	1606	34.27
88	Humulene epoxide II OS	1.27	–	–	–	1607	34.32
89	ND	–	–	–	0.64	1610	34.35
90	ND	0.25	–	–	–	1615	34.49
91	ND	0.13	0.16	–	0.53	1627	34.84
92	Alloaromadendrene epoxide	0.96	0.24	–	–	1633	35.00
93	Amorph-4-en-7-ol	–	–	–	0.11	1636	35.08
95	τ-Muurolol	3.06	0.72	–	–	1642	35.22
97	α-Cadinol	0.66	0.12	–	–	1655	35.62
98	8-Cedrene-13-ol	0.43	0.10	–	–	1661	35.77
99	ND	0.6	–	–	–	1675	36.16
100	Germacra-4(15),5,10(14)-trien-1-α-ol OSCAR	0.52	–	–	0.18	1690	36.586
102	ND	–	–	2.58	–	2109	46.72
	Total identified%	98.6	99.84	97.40	98.08		
	Monoterpene hydrocarbons	0.74	1.13	0.21	4.73		
	Oxygenated monoterpenes	20.5	71.92	73.25	51.89		
	Sesquiterpene hydrocarbons hydrocarbons	44.60	20.66	17.23	33.69		
	Oxygenated sesquiterpenes	32.54	6.11	5.75	5.73		
	others	0.22	0.16	0.95	2.04		

*RT* Retention time, *RI* Retention index, Content%’ in terms of peak area

In the EOs extracted from *A. citriodora* plants collected in Jerusalem, Hebron, and Qalqilya, 50, 32, and 30 compounds were identified, accounting for 98.8, 97.4, and 99.84% of the overall content of the EOs, respectively. Geranial (26.32–37.22%), neral (18.35–29.00%), and a-curcumene (7.76–16.91%) are the most prevalent components in these oils. Furthermore, the EO of *A. citriodora* from Jericho contained 32 chemicals, which accounted for 98.60% of the entire chromatographic area. The most prevalent of these were *a*-curcumene (26.94%), spathulenol (13.69%), geranial (10.79%), caryophyllene oxide (8.66%), and neral (7.59%). EO from Jerusalem contained significant amounts of limonene (4.06%), while both Hebron and Jerusalem EOs had noteworthy amounts of 1,8-cineole (2.70 and 3.96%, respectively). *β*-caryophellene was found in EOs from Hebron, Qalqilya, Jericho, and Jerusalem in concentrations of 1.54, 6.00, 6.14, and 7.95%, respectively. Four main categories generally classify the chemical constituents of essential oils: monoterpene hydrocarbons (MH), oxygenated monoterpenes (OM), sesquiterpene hydrocarbons (SH), and oxygenated sesquiterpenes (OS). The EO from Jerusalem had the highest concentration of MH (4.73%), while the EO from Hebron displayed the lowest value (0.21%). Hebron’s EO had the highest OM concentration (73.25%) and lacked OS, while Jericho’s EO primarily consisted of sesquiterpene compounds (77.14%), with 32.54% being oxygenated. In contrast, EOs from other regions contained lower concentrations of sesquiterpenes, ranging from 22.98 to 39.42%.

The observed variances in EOs can be attributed to unique climatic and geological conditions present at each of the four locations. Jericho is the lowest location on Earth, with summer temperatures exceeding 50 °C and winter temperatures not falling below 20 °C. Jericho is characterized by arid climates with very low humidity, and its soil and water have a significant salt content. Qalqilya, located in close proximity to the Mediterranean Sea, experiences elevated levels of humidity, with precipitation exceeding 900 mL. Jerusalem and Hebron are located in the central region of Palestine, notably on rugged topography. During the summer season, the temperature can reach a peak of 37 °C, while in winter, it can drop below the freezing point.

Aside from differences in the relative proportions of MH, OM, SH, and OS and their respective main components, each of the four EOs contains a number of unique chemicals. Except for those from Jericho, the tested EOs partially align with the existing literature data as geranial and neral are reported as the main components of EOs from *A. citriodora* [16, 20]. Furthermore, the bulk of academic papers on the EO compositions of *A. citriodora* Palau exhibit a continuous tendency, which is particularly highlighted by the prevalence of oxygenated monoterpenes. A few exceptions exist where OS dominates the profiles, such as spathulenol and caryophyllene oxide [19]. Geranial (26.8–38.3%), neral (20.8–29.6%), and limonene (5.7–20.6%) were discovered to be the most abundant components of *A. citriodora* EO from Portugal [20], whereas limonene, caryophyllene oxide, α-curcumene, geranial, and spathulenol were found to be the most abundant components of EO from Iran [23]. In their study, Karik et al. [24] found that the EO of lemon verbena primarily consists of geranial (22.7–35.8%), neral (15.6–26.6%), and limonene (12.2–31.4%). Additionally, smaller concentrations of *β*-caryophyllene (2.6–6.2%), caryophyllene oxide (3.0–6.0%), geranyl acetate (1.1–3.9%), spathulenol (2.5–4.9%), and ar-curcumene (1.5–5.6%) were detected.

Hosseini et al. [19] found eugenol (14.63%), D-limonene (12.41%), caryophyllene oxide (8.78%), *α*-curcumene (7.91%), geranial (7.44%), *β*-spathulenol (6.92%), neral (5.38%), and eucalyptol (5.3%) were the major components of EO lemon verbena growing in Iran. According to Benelli et al. [25], the main components of Italian lemon verbena EO were geranial (21%), neral (16.5%), limonene (11.4%), 1,8-cineole (7%), *α*-curcumene (6.7%), spathulenol (5.8%), and caryophyllene oxide (4.8%). The chemical analysis of EO obtained from Agadir, Morocco, indicated that caryophyllene oxide (13.68%), spathulenol (12.38%), and *α*-curcumene (12.32%) were the major constituents [26]. EO from Argentina, on the other hand, was discovered to be high in myrcenone (36.50%), α-thujone (13.10%), lippifoli-1(6)-en-5-one (8.87%), and limonene (6.87%) [27]. The EO from Turkey contains citrals in the range of 17.90–27.10% and limonene in the range of 14.8–18.6% [28]. The chemical composition of EOs is well acknowledged to be altered by geographical region, climate, humidity, harvesting season, and extraction procedure.

### Anticancer

Despite recent progress in our understanding of the molecular and biological aspects of colon cancer, this illness continues to be a major contributor to mortality [29]. The urgent need to investigate new and effective therapeutic options arises from the high incidence of medication resistance, particularly in advanced stages of disease. Colon cancer is widely recognized for its elevated expression of matrix metalloproteinase-9 (MMP-9), an enzyme that plays a critical role in the formation, growth, and development of adenomas [30]. In contrast to the MMP9 gene, the KLF2 gene functions as a tumour suppressor, inhibiting the growth and metastasis of cancer [31, 32]. Medicinal plants’ active ingredients, especially those found in essential oils, have a significant impact on the development of cancer. It increases apoptosis and reduces drug resistance in a variety of malignancies [7, 13, 33].

To the best of our current understanding, this study represents the first comprehensive attempt to assess the effectiveness of the aerial parts of *A. citriodora* from four different regions in Palestine in inhibiting the growth of colon cancer cells.

**Fig. 2:**
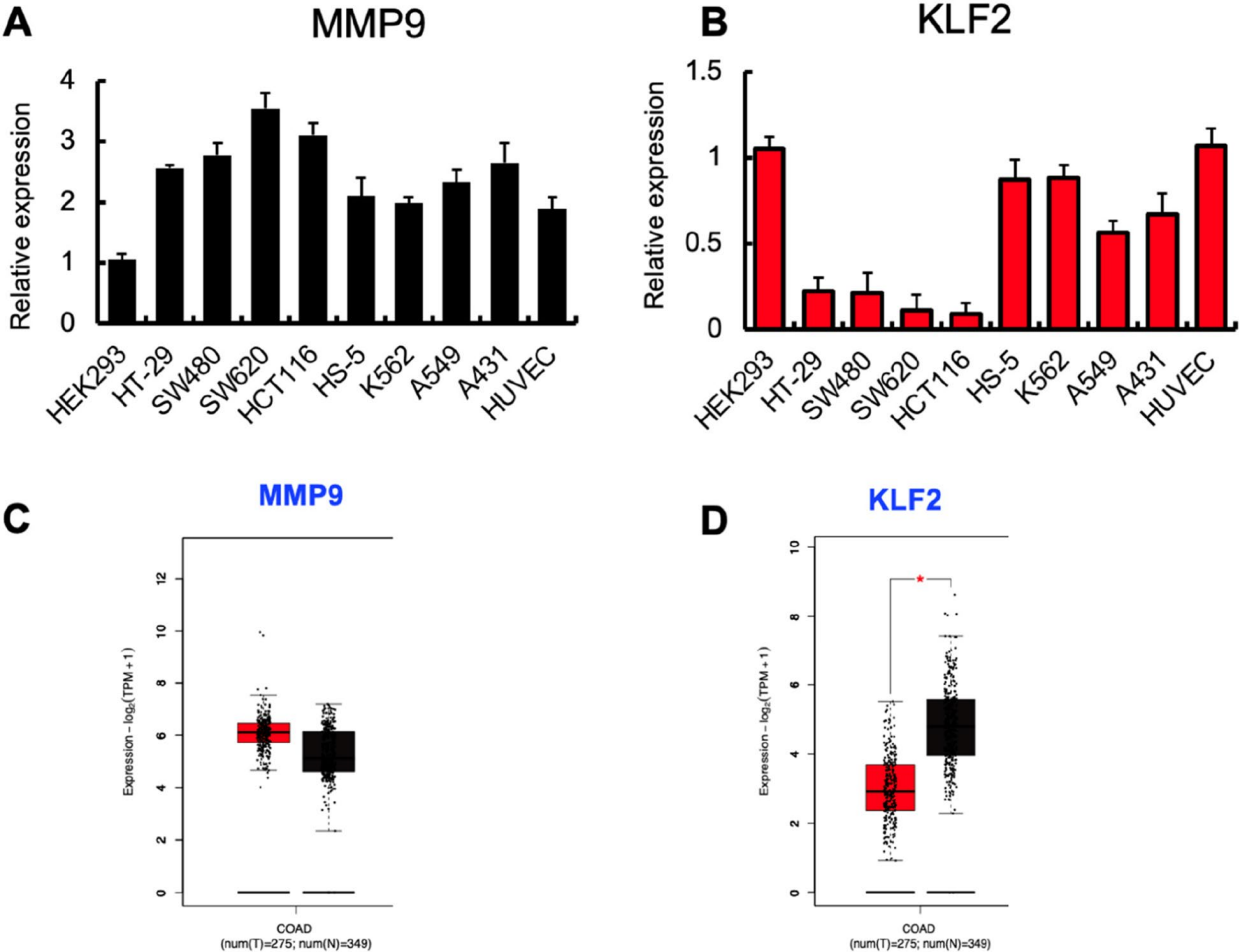
**A** and **B** Human HEK293, HT-29, SW480, SW620, HCT116, HS-5, K562, A549 and HUVEC cells were analysed for MMP9 and KLF2 mRNA expression by qPCR (*n* = 3/ cell line). Expression was compared to the expression in HEK293 cells. **C** and **D** Expression levels of human MMP9 and KLF2 in tumors (T) and adjacent normal tissues (N) taken from COAD patients. Data were obtained from the GEPIA 2 database

The quantitative polymerase chain reaction (qPCR) analysis was performed using RNA samples isolated from both colon and non-colon cancer cell lines. The results demonstrated a noteworthy increase in the expression of MMP9 (Fig. 2A) and a decrease in the expression of KLf2 (Fig. 2B) in four separate types of colon cancer cells. These findings are consistent with data from GEPIA 2, an open human cancer database (Fig. 2C, D) [21].

**Fig. 3:**
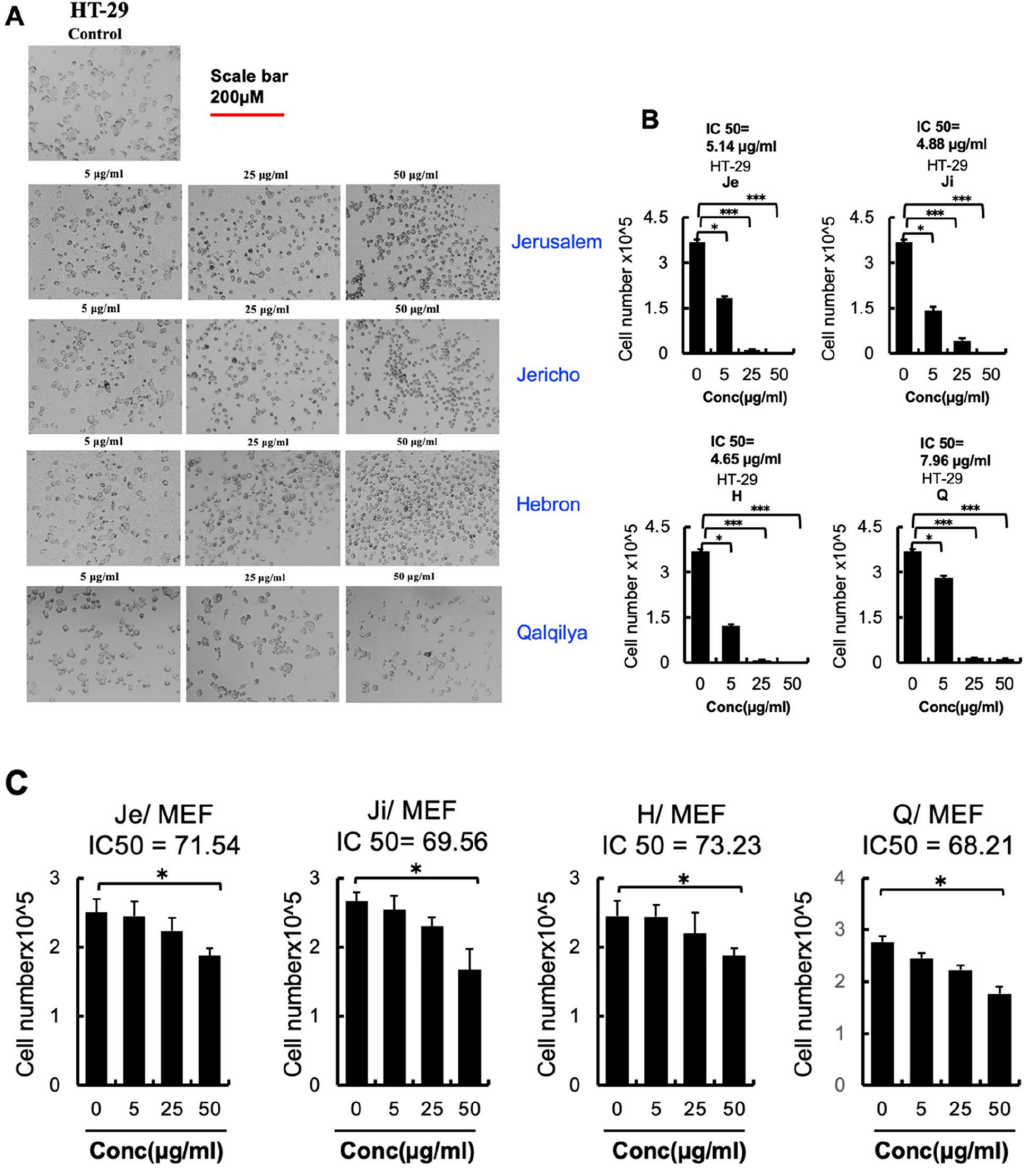
**A** Macroscopic images of HT-29 cells after 24 hours of AC-EO treatment or DMSO as a control (*n* = 3 groups). Scale bar: 200 μM. The indicated doses of AC-EO were applied to HT-29 cells in (**B**). After 24 hours treatment (*n* = 6). Viable cells were counted using Trypan blue after 24 h (*n* = 6/group). **C** MEF were treated with indicated dose of EO, after 24 hours viable cells were counted using trypan blue (*n* = 6/group). Data are expressed as mean +/− SEM. **p 0.01; *p 0.05 (Student’s t-test)

The effect of different doses of *A. citriodora* EOs on the proliferation of HT-29 human colon cancer cells in vitro using MTT cell proliferation assay was investigated. After a 24-hour treatment with EO, the proliferative ability of HT-29 cells was reduced in a concentration-dependent manner, with IC_50_ values of 5.14, 4.88, 4.65 and 7.96 μg/mL for Jerusalem, Jericho, Hebron, and Qalqilya EO, respectively (Fig. 3A and B). We noticed EO from Qalqilya was less effective against HT-29 cells compared to other three EOs. DMSO carrier treatment had no effect on cell proliferation (Fig. 3B). A dose of 50 μg/mL of EO demonstrated complete suppression of tumour development.

**Fig. 4:**
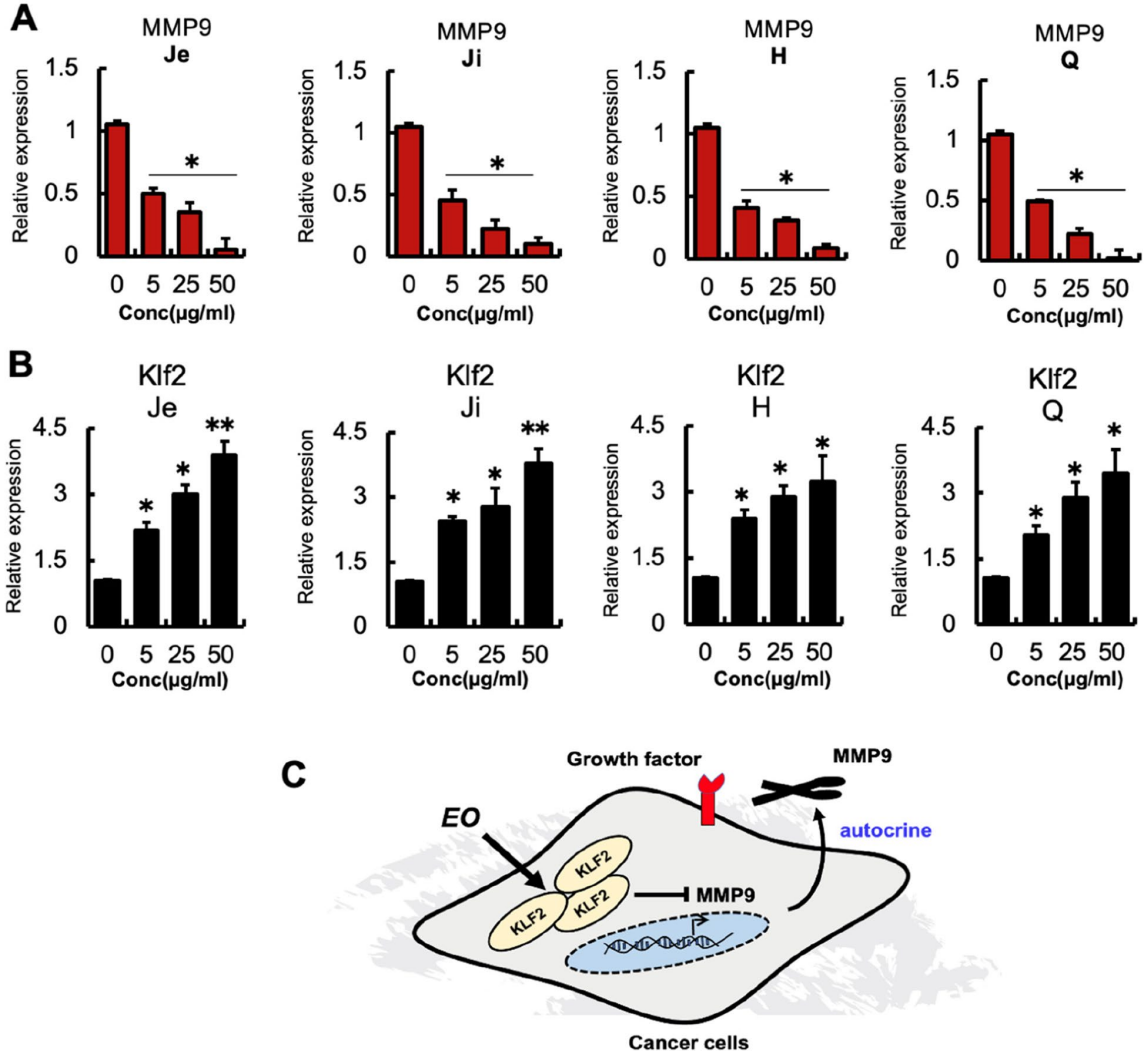
**A** and **B** Human HT-29 cells were analyzed after EO treatment at the indicated concentration for MMP9 and KLF2 mRNA expression by qPCR (*n* = 3/ cell line). Expression was compared to control DMSO group (0 μg/ml). Data are expressed as mean +/− SD. **p 0.01; *p 0.05 (Student’s t-test). **C** hypothesized mechanism by which EO inhibits HT-29 cell growth by upregulating KLF2 and inhibiting MMP9. Abbreviations: MMP9, Matrix metalloprotrinase-9; EO, essential oil; KLF2, Kruppel-like transcription factor 2

Cytotoxicity is an anti-cancer drug’s dose-limiting toxicity. Previous research demonstrated that mouse embryonic fibroblasts (MEF-1) can be used to assess non-malignant cell toxicity [2]. When up to 25 μg/ml of AC-EO was added (Fig. 3C), MEF-1 did not exhibit a reduction in proliferation. Based on these data, it can be concluded that *A. citriodora* EO enhanced the anti-proliferative effects on cancer cells, thereby reducing drug dosage and minimizing cytotoxicity. Quantitative polymerase chain reaction (qPCR) analysis confirmed these findings by showing a reduction in MMP9 expression in HT-29 cells after treatment with EOs from four distinct areas compared to the control group (Fig. 4A). Furthermore, it was noted that there was an increase in the expression of KLF2 under the same experimental conditions, as depicted in (Fig. 4B). Chemical composition of EO derived from the aerial leaves of *A. citriodora* Palau which isolated from Palestine shared common active ingredients which has significant role in downregulating MMP9 and enhancing KLf2 expression as others reported previously. These data imply that AC-EO induces upregulation of KLF2 and downregulate MMP9 in vitro and in vivo (Fig. 4C). Our findings are consistent with those of Mileni et al. [34], who found that extract from *Aloysia polystachya* induces the Cell Death of Colorectal Cancer Stem Cells and Nandan et al., who showed that KLF2 suppresses MMP9 expression in human retinoblastoma [35].

### Antimicrobial activity

The study employed the broth microdilution assay to determine the MIC of *A. citriodora* EOs against six bacterial strains and one fungal strain. The inhibitory activity of the EOs was evaluated, and the results are presented in Table 2. The e *A. citriodora* EO from Jerusalem demonstrated a significant inhibitory impact on MRSA and *S. aureus*, with a MIC value of 0.39 ± 0.02 mg/mL. Conversely, the EO derived from Jericho demonstrates a modest level of antibacterial activity against MRSA and *S. aureus*, with a MIC value of 1.56 ± 0.03 mg/mL. Conversely, the other EOs possess a somewhat lower antimicrobial efficacy. The EOs that were examined showed exceptional antibacterial effectiveness against both MRSA compared to the positive control (ciprofloxacin) (Table 2). However, both *E. coli*, *K. pneumonia,* and *P. aeruginosa* exhibited resistance to all tested EOs. EO originating from Qalqilya exhibited the lowest antibacterial efficacy. *A. citriodora* EOs from Jerusalem and Jericho exhibited potent antifungal properties against *C. albicans*, with MIC value of 0.048 ± 0.01 mg/mL, surpassing the antifungal medication Fluconazole’s MIC value of 1.56 ± 0.1 mg/mL. The MIC of the *A. citriodora* EO was comparable to that of the positive control. However, the EO from Qalqilya exhibited lesser antifungal activity, with a MIC of 3.125 ± 0.3 mg/mL. Differences in the chemical composition of EOs from four different sites in Palestine account for the discrepancies in their antibacterial effectiveness. The EOs obtained from Jericho and Jerusalem contains notable concentrations of α-curcumene, measuring 16.91 and 26.74%, respectively. Additionally, it is noteworthy to mention that Jericho EO contains substantial amounts of spathulenol and caryophellene oxide, which account for around 13.69 and 8.66% of its overall composition, respectively. The results of this study partially align with previous research that has shown the antibacterial characteristics of *A. citriodora* EO. According to a study conducted by Hosseini et al. [23], EO extracted from Iranian lemon verbena had a moderate bactericidal effect against all bacteria tested, including 4 gram-negative bacteria (*E. coli*, *S. typhimurium*, *P. aeruginosa*, and *S. dysenteriae*) and 3 gram-positive bacteria (*S. aureus*, *L. monocytogenes*, and *B. cereus*). The MIC values for *S. aureus*, *E. coli*, and *P. aeruginosa* were found to be 1250, 2500, and 2500 μg/mL, respectively [15]. Oukerrou et al. discovered that EOs obtained from *A. citriodora* plants gathered from several places in Morocco demonstrated a low degree of antibacterial effectiveness. The MIC values against *E. coli* exhibited a range of 2.84 to 8.37 mg/mL, whereas the values against *S. aureus* ranged from 7.43 to 12.12 mg/mL [26]. Researchers tested the antimicrobial efficacy of the EO of *A. citriodora* grown in Greece against seven different types of common food spoilage and pathogenic bacteria. They found that it had a modest bactericidal effect against *E. coli* and *S. aureus*, with MIC values of 7024 ± 9 and 6901 ± 18 mg/mL, respectively [16]. On the other hand, *A. citriodora* essential oils showed considerable antifungal activity against *Candida* species, with inhibitory diameter zones ranging from 858.04 to 858.27 mm against *C. albicans* (ATCC 2091).

**Table 2:** Antimicrobial effects MIC (mg/mL) of *A. citriodora* EO and positive controls

	Bacteria						Fungus
	Gram-positive		Gram-Negative				Yeast
**ATCC Number/strain**	Clinical strain	ATCC 25923	ATCC 25922	ATCC 13883	ATCC 8427	ATCC 9027	ATCC 90028
**Microbe**	MRSA	*S. aureus*	*E. coli*	*K. pneumoniae*	*P. vulgaris*	*P. aeruginosa*	*C. albican*
**Hebron EO**	3.125 ± 0.1	3.125 ± 0.2	12.5 ± 0.78	12.5 ± 0.60	3.125 ± 0.40	25 ± 0.85	1.56 ± 0.10
**Jerusalem EO**	0.39 ± 0.02	0.39 ± 0.03	12.5 ± 0.4	12.5 ± 0.91	3.125 ± 0.1	R	0.048 ± 0.01
**Jericho EO**	1.56 ± 0.05	1.56 ± 0.06	25 ± 0.70	25 ± 0.69	12.5 ± 0.75	25 ± 0.93	0.048 ± 0.01
**Qalqilya EO**	3.125 ± 0.2	3.125 ± 0.3	25 ± 1.2	6.25 ± 0.70	25 ± 1.2	12.75	3.125 ± 0.3
**Ciprofloxacin**	12.5 ± 0.09	0.78 ± 0.01	1.56 ± 0.1	0.13 ± 0.01	15 ± 1.1	3.12 ± 0.35	R
**Ampicillin**	25 ± 0.10	25 ± 0.125	3.12 ± 0.35	1.25 ± 0.1	18 ± 1.04	R	R
**Fluconazole**	R	R	R	R	R	R	1.56 ± 0.1

Where *R* Resistance

As illustrated in Table 2, the MIC values revealed that Jerusalem EO, followed by Jericho EO, were the most active against *S. aureus* and MRSA. In contrast, Jerusalem and Hebron EOs were the most active against *P. vulgaris*. Qalqilya EO was the most effective against *K. pneumonia*. All four EOs evaluated exhibited little or no efficacy against *P. aeruginosa*. Previous research has indicated that this particular bacterial strain possesses inherent resistance to many types of EOs. The qualitative and quantitative variety of the chemical components present in these EOs, as well as their distinct molecular mechanisms, explain the disparity in antibacterial activity of the tested EOs. Because EOs contain a lot of different chemicals, it seems more likely that their ability to kill bacteria is due to a number of different mechanisms working together on different cell targets [13]. Indeed, among the main compounds in the four essential oils investigated, spathulenol, *β*-caryophyllene, caryophyllene oxide, and 1,8-cineole show antibacterial and antifungal properties [36, 37], while citral (neral and geranial) has a high antibacterial impact [38]. The interaction of *p*-cymene, *γ*-terpinene, and phenolic compounds may have antibacterial properties [39]. Synergism between constituents can have a significantly greater impact than the expected activity of the principal chemicals [9].

## Conclusions

Geranial, neral, and *α*-curcumene were found in varying proportions in the EOs collected from Qalqilya, Hebron, and Jerusalem, whereas α-curcumene, spathulenol, geranial, caryophyllene oxide, and neral were the principal components of EO from Jericho. The findings of the present study reveal that the *A. citriodora* EOs collected from four distinct places in Palestine had strong anticancer activities. Through the upregulation of the tumor suppressor gene KLF2, EOs from four different regions reduced the development and proliferation of colon cancer cells and the production of MMP9. The EOs of *A. citrodora* from Jericho and Jerusalem strongly inhibited the *C. albican*, while only EO from Jerusalem and showed potent antibacterial activity against MRSA and *S. aureus*. These findings highlight the significant potential of *A. citriodora* EO from Jerusalem and Jericho as a powerful natural agent for treating disorders associated with melanoma cancer and eradicating *C. albican.* As a result, further research into the molecular processes by which EOs act in vivo is required to validate and establish their efficacy for possible application in cancer treatment.

## Data Availability

No datasets were generated or analysed during the current study.
